# Sex and gender differences in nutrition research: considerations with the transgender and gender nonconforming population

**DOI:** 10.1186/s12937-021-00662-z

**Published:** 2021-01-15

**Authors:** Whitney Linsenmeyer, Jennifer Waters

**Affiliations:** 1 Doisy College of Health Sciences, Saint Louis University, 3437 Caroline St, St. Louis, MO 63104 USA; 2 Rush Copley Medical Center, 2000 Ogden Avenue, Aurora, IL 60504 USA

**Keywords:** Transgender, Gender nonconforming, Nutrition assessment

## Abstract

A sex- and gender-informed approach to study design, analysis and reporting has particular relevance to the transgender and gender nonconforming population (TGNC) where sex and gender identity differ. Notable research gaps persist related to dietary intake, validity and reliability of nutrition assessment methods, and nutrition interventions with TGNC populations. This is due in part to the conflation of sex and gender into one binary category (male or female) in many nutrition surveillance programs worldwide. Adoption of the Sex and Gender Equity In Research (SAGER) guidelines and the two-step method of querying sex and gender has the potential to exponentially increase the body of research related to TGNC health.

In this special issue on sex and gender differences in dietary intake and other dietary behaviors across the life course, we wish to bring to the table certain considerations regarding the transgender and gender nonconforming (TGNC) population.

Whereas sex refers to the biological attributes of females and males, gender refers to the socially constructed roles and behaviors associated with a feminine, masculine, or non-binary identity. The constructs of sex and gender are separate health determinants, and yet historically these variables have either been conflated or omitted entirely. Thus, a sex- and gender-informed approach to study design, analysis and reporting has great potential to increase the rigor and the relevance of scientific research [1].

This movement has particular relevance to the TGNC population where sex and gender identity differ. The term transgender may be used to describe a person whose gender identity differs from the sex that was assigned at birth, and the term gender nonconforming or genderqueer may be used to describe a person whose gender identity exists in a more fluid or multifaceted manner [2]. Worldwide estimates of TGNC demographics have been notoriously difficult to obtain given the variability in terminology, gender-based stigma and discrimination in many cultures, and respondents’ concerns regarding confidentiality and anonymity; current estimates include 0.6 % of the adult population in the United States, a figure that has doubled in the past decade [3, 4].

Nutrition-related considerations for the TGNC population are both clinical and psychosocial in nature. Those undergoing masculinizing or feminizing hormone therapy may experience weight gain, changes in body composition, altered lipid profiles, and changes in bone composition. Existing research also points to elevated rates of eating disorders, food insecurity, and discrimination both outside of and related to the medical community [5, 6]. A recent scoping review identified notable research gaps related to dietary intake, validity and reliability of nutrition assessment methods, and nutrition interventions with TGNC populations [7].

Designing studies to address the identified research gaps may present significant challenges. Many large-scale health surveillance databases frequently utilized by researchers to study the dietary behaviors and nutritional status of various groups of people are not as useful for studying the TGNC population due to the conflation of sex and gender into one binary category (male or female). This is observed in the United States with the National Health and Nutrition Examination Survey (NHANES) and the National Health Interview Survey (NHIS), as well as other nutrition surveillance programs throughout the world including the Canadian Health Survey (CHS), Australian Health Survey (AHS), and China Health and Nutrition Survey (CHNS).

The Sex and Gender Equity In Research (SAGER) guidelines offer researchers an approach to standardize reporting of sex and gender for research purposes. The guidelines endorse clear a distinction between sex and gender, as well as an analysis of sex and gender differences and similarities where appropriate [8]. When TGNC identities are included in this approach, commonly referred to as the two-step method, researchers have the opportunity to stratify data based on sex and a range of gender identities beyond male and female (Fig. 1) [9].

**Fig. 1:**
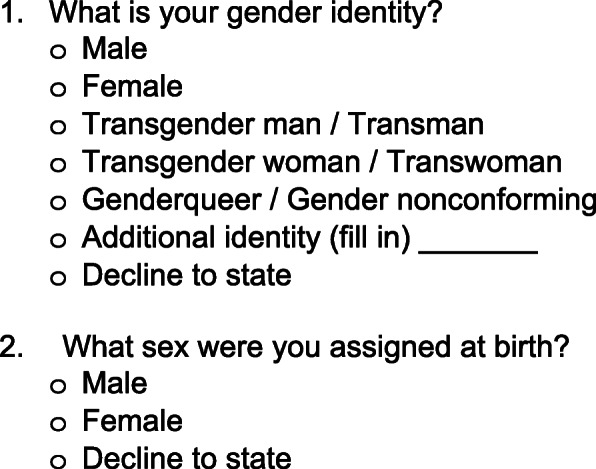
Two-step method for the collection of sex and gender identity data

Adoption of this approach in nutrition surveillance programs and other systematic research could help to bridge the literature gaps on a range of topics impacting the TGNC population including dietary intake and eating behaviors, utilization of food and nutrition assistance programs, food access and security, weight history, engagement in physical activity, and consumption of dietary supplements. Ultimately, a sex- and gender-informed approach within systematic research has the potential to exponentially expand the body of research related to the health of the TGNC population.

## Data Availability

Not Applicable. Not Applicable. Not Applicable. The authors declare that they have no competing interests.

## Abbreviations

TGNC: Transgender and gender nonconforming
NHANES: National Health and Nutrition Examination Survey
NHIS: National Health Interview Survey
CHS: Canadian Health Survey
AHS: Australian Health Survey
CHNS: China Health and Nutrition Survey
SAGER: Sex and Gender Equity In Research
